# Supplementary material for: A Lipidomics- and Transcriptomics-Based Analysis of the Intestine of Genetically Obese (ob/ob) and Diabetic (db/db) Mice: Links with Inflammation and Gut Microbiota
Source: Cells. 2023 Jan 25;12(3):411. doi: 10.3390/cells12030411 (PMC9913869; doi:10.3390/cells12030411)
Supplement: Supplementary file 1 [file cells-12-00411-s001.zip › cells-2140044-supplementary.pdf]

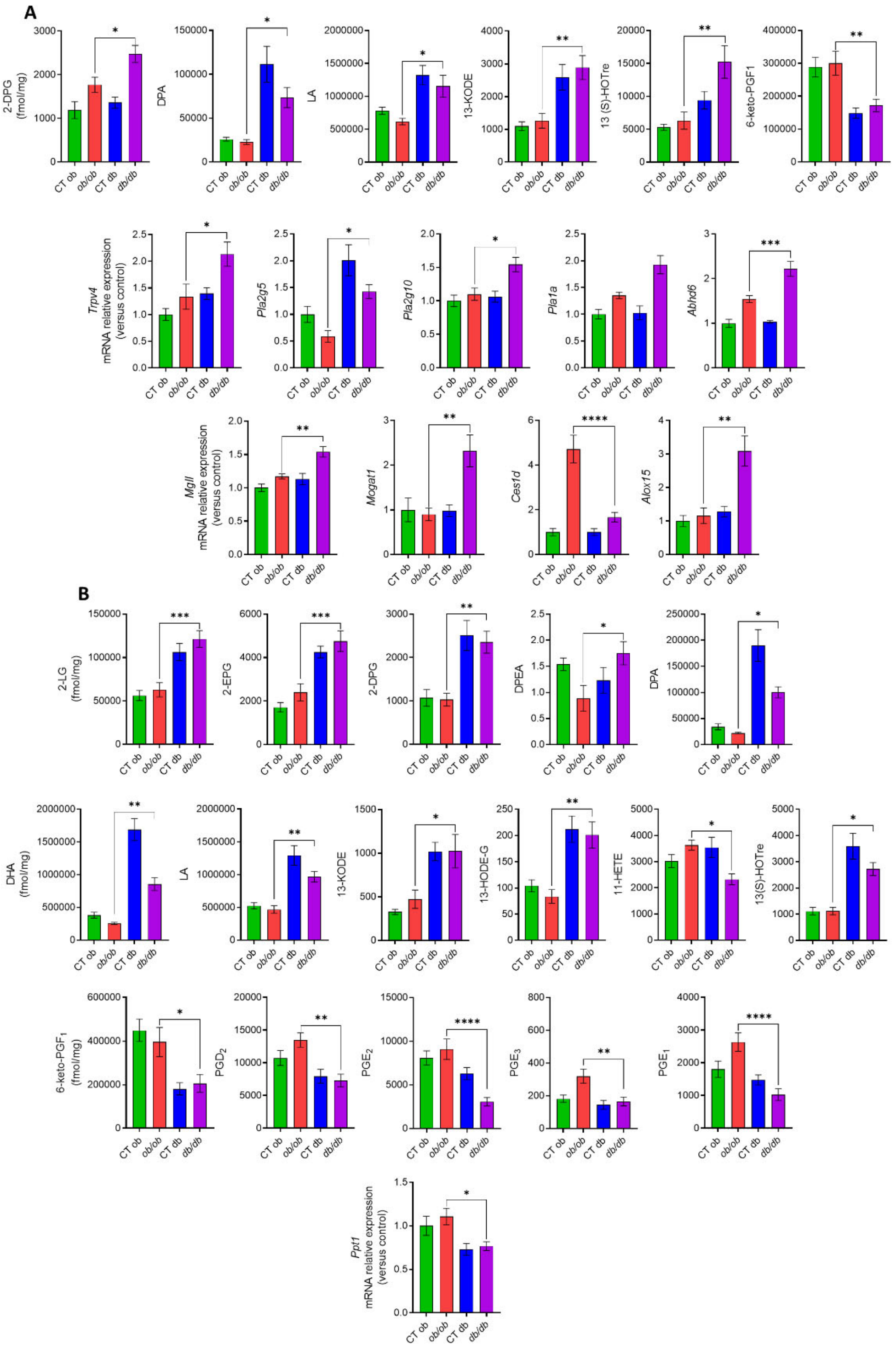

Supplementary Figure S1. Different eCBome tone in the jejunum and the ileum of ob/ob and db/db mice. (**A**) Concentrations of the eCBome-related mediators in the jejunum and ileum, respectively (fmol/mg wet tissue weight), measured by HPLC-MS/MS. (**B**) mRNA expression of receptors and metabolic enzymes for 2-monoacylglycerols and N-acylethanolamines in the jejunum and the ileum, respectively, measured by qPCR-based TaqMan Open Array. Data are presented as the mean ± S.E.M of n=7-10. * P ≤ 0.05, **P ≤ 0.01, ***P ≤ 0.005, ****P ≤ 0.001. For mRNA expression, relative units were calculated versus the mean of the CT ob mice values set at 1. Data were analyzed by one-way ANOVA followed by Tukey's post hoc test. Abbreviations: see supplementary Table S1 and Table S2.

Supplementary Table S1. Endocannabinoid and related lipid mediator abbreviations.

Symbol	Endocannabinoids name
**2-AG**	1(3)- and 2-arachidonoyl-glycerol
**AEA**	*N*-arachidonoyl-ethanolamine
**DHEA**	*N*-docosahexanoyl-ethanolamine
**2-DHG**	1(3)- and 2-docosahexaenoyl-glycerol
**DPA**	docosapentaenoic acid (n-3)
**2-DPG**	1(3)- and 2-docospentaenoyl-glycerol (n-3)
**EPEA**	*N*-eicosapentanoyl-ethanolamine
**EPA**	eicosapentaenoic acid
**2-EPG**	2-eicosapentaenoyl-glycerol
**15-HEPE**	15-hydroxyeicosapentaenoic acid
**18-HEPE**	18-hydroxyeicosapentaenoic acid
**13-HODE**	13-hydroxyoctadecadienoic acid
**13-HODE-G**	13-hydroxyoctadecadienoyl-glycerol
**11-HETE**	11-hydroxyeicosatetraenoic acid
**13(S)-HOTre**	13S-hydroxy-9Z,11E,15Z-octadecatrienoic acid
**12-HEPE**	12-hydroxyeicosapentaenoic acid
**15-HEPE**	15-hydroxyeicosapentaenoic acid
**14-HDHA**	14-hydroxydocosahexaenoic acid
**17-HDHA**	14-hydroxydocosahexaenoic acid
**LA**	Linoleic acid
**LEA**	*N*-linoleoyl-ethanolamine
**2-LG**	1(3)- and 2-linoleoyl-glycerol
**SEA**	*N*-stearoyl-ethanolamine
**OEA**	*N*-oleoyl-ethanolamine
**2-OG**	1(3)- and 2-oleoyl-glycerol
**PEA**	*N*-palmitoyl-ethanolamine
**2-PG**	2-palmitoyl-glycerol
**NAGly**	*N*-arachidonoyl glycine
**PGD_2**	Prostaglandin D_2
**PGE_2**	Prostaglandin E_2

Supplementary Table S2. List of the genes analyzed by qPCR based TaqMan Open Array and their function.

Gene symbol	Gene name	Function
Cacna1b	calcium channel, voltage-dependent, T type, alpha 1B subunit	receptor
Cacna1h	calcium channel, voltage-dependent, T type, alpha 1H subunit	receptor
Cnr1	cannabinoid receptor 1	receptor
Cnr2	cannabinoid receptor 2	receptor
Gpr119	G protein-coupled receptor 119	receptor
Gpr18	G protein-coupled receptor 18	receptor
Gpr55	G protein-coupled receptor 55	receptor
Ppara	peroxisome proliferator activated receptor alpha	receptor
Pparg	peroxisome proliferator activated receptor gamma	receptor
Ptgfr	prostaglandin F receptor	receptor
Trpv1	transient receptor potential cation channel, subfamily V, member 1	ligand-activated channel
Trpv2	transient receptor potential cation channel, subfamily V, member 2	ligand-activated channel
Trpv4	transient receptor potential cation channel, subfamily V, member 4	ligand-activated channel
Abhd4	abhydrolase domain containing 4	anabolic enzyme for NAEs
Akr1b3	aldo-keto reductase family 1, member B3 (aldose reductase)	anabolic enzyme for prostamides, catabolic enzyme for AEA and 2-AG
Fam213b	family with sequence similarity 213, member B	anabolic enzyme for prostamides, catabolic enzyme for AEA and 2-AG
Gde1	glycerophosphodiester phosphodiesterase 1	anabolic enzyme for NAEs
Gdpd1	glycerophosphodiester phosphodiesterase domain containing 1	anabolic enzyme for NAEs
Hrasls5	HRAS-like suppressor family, member 5	anabolic enzyme for NAEs
Inpp5d	inositol polyphosphate-5-phosphatase D	anabolic enzyme for NAEs
Napepld	N-acyl phosphatidylethanolamine-specific phospholipase D-like enzyme	anabolic enzyme for NAEs
Pla2g5	phospholipase A2, group V	AA-releasing enzyme possibly involved in phospholipid remodeling and hence biosynthesis of eCB precursors
Ptgs2	prostaglandin-endoperoxide synthase 2	anabolic enzyme for prostamides, catabolic enzyme for AEA and 2-AG
Ptpn22	protein tyrosine phosphatase, non-receptor type 22 (lymphoid)	anabolic enzyme for AEA
Ptges	prostaglandin E synthase	anabolic enzyme for prostamides, catabolic enzyme for AEA and 2-AG
Comt	catechol-O-methyltransferase	catabolic enzyme for *N*-acyl-dopamines
Faah	fatty acid amide hydrolase	catabolic enzyme for NAEs, primary fatty acid amides, *N*-acyl-taurines and *N*-acyl-glycines
Naaa	N-acylethanolamine acid amidase	catabolic enzyme for saturated NAEs
Pam	peptidylglycine alpha-amidating monooxygenase	anabolic enzyme for primary fatty acid amides, catabolic enzyme for *N*-acyl-glycines
Dagla	diacylglycerol lipase, alpha	anabolic enzyme for 2-acylglycerols

Daglb	diacylglycerol lipase, beta	anabolic enzyme for 2-acylglycerols
Dgke	diacylglycerol kinase, epsilon	anabolic/catabolic enzyme for 2-acylglycerols
Enpp2	ectonucleotide pyrophosphatase/phosphodiesterase 2	autotaxin- a LysoPLD: produces LPA.
Pla1a	phospholipase A1 member A	anabolic enzyme for 2-acylglycerols
Plcb1	phospholipase C, beta 1	anabolic enzyme for 2-acylglycerols
Abhd12	abhydrolase domain containing 12	catabolic enzyme for monocylglycerols
Abhd16a	abhydrolase domain containing 16	catabolic enzyme for monocylglycerols
Abhd6	abhydrolase domain containing 6	catabolic enzyme for monocylglycerols
Agk	acylglycerol kinase	catabolic enzyme for monocylglycerols
Alox12	arachidonate 12-lipoxygenase	catabolic enzyme for AEA and 2-AG
Alox15	arachidonate 15-lipoxygenase	catabolic enzyme for AEA and 2-AG
Ces1d	carboxylesterase 1D	catabolic enzyme for monoacylglycerols
Ces2h	carboxylesterase 2H	catabolic enzyme for monoacylglycerols
Mgll (Magl)	monoglyceride lipase	catabolic enzyme for monoacylglycerols
Mogat1	monoacylglycerol O-acyltransferase 1	catabolic enzyme for monoacylglycerols
Ppt1	palmitoyl-protein thioesterase 1	catabolic enzyme for 2-AG
Gapdh	glyceraldehyde-3-phosphate dehydrogenase	reference gene
Hprt	hypoxanthine guanine phosphoribosyl transferase	reference gene
Rps13	ribosomal protein S13	reference gene
Tbp	TATA box binding protein	reference gene

Supplementary Table S3. qPCR primer sequences for the targeted mouse genes.

Gene symbol	Gene name	Forward Primer sequence (5'-3')	Reverse Primer sequence (5'-3')
Rorgt2	Retinoid orphan receptor gamma t	GAAATGTTGTTGACCCCCTCT	TCATACCCAAACCCCATAAAAC
Il17a	Interleukin 17a	AAGGCCCTCAGACTACCTCAA	TTCCCAGATCACAGAGGGATATCTA
Rpl19	Ribosomal Protein L19	GAAGGTCAAAGGGAATGTGTTCA	CCTTGTCTGCCTTCAGCTTGT
